# Another Set of Eyes: Recipients’ Views of the Benefits of Geriatric Specialty Telehealth Services

**DOI:** 10.1093/geroni/igab046.456

**Published:** 2021-12-17

**Authors:** Eileen Dryden, Laura Kernan, Kathryn Nearing, Camilla Pimentel, Lauren Moo

**Affiliations:** 1 Veterans Health Administration, Bedford, Massachusetts, United States; 2 Veterans Health Administration, Denver, Colorado, United States; 3 VA Bedford Healthcare System, Bedford, Massachusetts, United States; 4 VA Bedford Health Care System, Bedford, Massachusetts, United States

## Abstract

The aim of GRECC Connect is to increase access to specialty care for medically complex, older, rural patients through e-consultations and telehealth visits. We interviewed 50 outpatient clinic staff and providers as well as 30 patients and caregivers about these services. Overall, the services were considered beneficial. For patients and caregivers, services alleviated the stress and cost of travel, they improved quality of life by increasing their understanding of the progression of an illness and providing treatment and guidance to increase patient functioning and reduce disruptive behaviors, and they eased anxiety associated with not receiving needed care. Having ‘another set of eyes’ on the patients reduced stress and anxiety for providers. Concerns included alignment of telehealth modality with the capabilities of older patients with cognitive problems, hearing loss and/or limited technological abilities and, for some providers, that the referral for and recommendations resulting from the service added to their workload.

